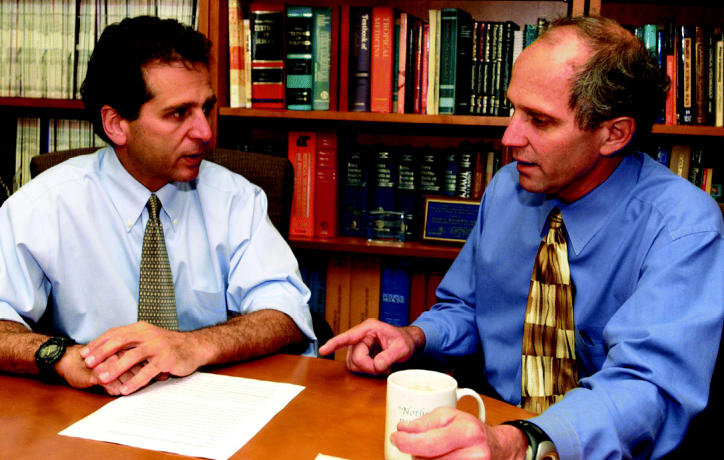# The NIEHS and the National Toxicology Program: An Integrated Scientific Vision

**DOI:** 10.1289/ehp.113-1257658

**Published:** 2005-07

**Authors:** Christopher J. Portier, David A. Schwartz

**Affiliations:** Associate Director, NTP E-mail: portier@niehs.nih.gov; Director, NIEHS and NTP E-mail: schwartzd@niehs.nih.gov

The National Toxicology Program (NTP) is an interagency program^1^ whose mission is to coordinate, conduct, and communicate toxicological research findings across the U.S. government. The NTP is administratively housed at the NIEHS, and David Schwartz serves as the director of both the NTP and the NIEHS. The NTP and the NIEHS share an integrated vision that serves to enhance the productivity of each program by promoting extensive collaboration across the broad spectrum of environmental health sciences. One of the emerging challenges for the NTP and the NIEHS is to use the best science to create, validate, and implement in environmental health research novel, robust, and efficient biological assays that will more effectively predict the risk of human disease and protect the health of our public.

Recently the NTP developed a vision statement, “Toxicology in the 21st Century: The Role of the National Toxicology Program,” and a roadmap for implementing this vision (available at http://ntp-server.niehs.nih.gov/). The NTP vision is to develop and use the best science possible to achieve a greater understanding of the mechanisms of toxicity, and to apply this understanding to the study of a broad array of environmental agents through the most effective and efficient use of resources. This vision is consistent with the need of the NIEHS to identify and understand the human biologic and pathophysiologic response to toxicants. As we begin to develop a strategic plan for the NIEHS, an understanding of the NTP vision and roadmap can help to inform and guide this process.

The NTP roadmap has identified four key scientific areas as priorities. First, the NTP needs to modernize the mammalian assays used to screen for toxicity. NIEHS research will lead the development of such assays. Critical to this process is increasing our understanding of the similarities and differences between laboratory species and humans. It is clear that both the quality and the interpretability of toxicity data will improve through the strategic use of new approaches to comparative biology.

Second, the NTP needs to develop and implement high- and medium-throughput screens to identify and understand potential targets for environmentally mediated disease. Such screens, ranging in complexity from simple subcellular fractions to complicated mixtures of primary cultures, can address a variety of biochemical, mechanistic, and functional end points. The availability of these screens will allow the NTP to establish priorities for full-scale, resource-intensive mammalian assays, and will provide direct links into the hypothesis-driven research supported by the NIEHS. As part of this process, the NTP and the NIEHS are collaborating with investigators in the NIH Roadmap Molecular Libraries and Imaging Initiative who are screening more than 100,000 compounds against multiple cellular targets to identify possible therapeutic agents and basic biologic responses. The *in vivo* toxicity data in the NTP archives will be incorporated into this high-throughput screen, and will serve as a cornerstone for the NIEHS and others to develop linkages between basic biologic responses and pathophysiologic outcomes.

Third, recent developments in biomedical research and molecular genetics have created a tremendous need to develop better ways to store, retrieve, analyze, and interpret vast amounts of data. The need for databases and repositories is critical for evaluating the toxicity of potentially hazardous agents. The NTP and the NIEHS will play important and complementary roles in developing these resources, and will partner with others to develop similar tools for the wider range of toxicologic, biologic, genetic, genomic, and biochemical information.

Finally, training the next generation of scientists is critical to the environmental health sciences. In collaboration with the NTP member agencies and our colleagues at the National Institutes of Health, we will support the development of training programs focused on creating integrated teams of scientists to understand and attack environmental health problems of concern to the public.

The NTP and the NIEHS share an integrated vision that serves to enhance the productivity of each program by promoting extensive collaboration across the broad spectrum of environmental health sciences.

The fields of toxicology and environmental health sciences are intimately linked, and the future for both is challenging. We are excited by the possibilities posed by the new directions of the NTP and the NIEHS, and look forward to the continued evolution of this vital and productive relationship.

## Figures and Tables

**Figure:**